# Baicalin Modulates Glycolysis *via* the PKC/Raf/MEK/ERK and PI3K/AKT Signaling Pathways to Attenuate IFN-I-Induced Neutrophil NETosis

**DOI:** 10.1155/mi/8822728

**Published:** 2025-05-19

**Authors:** Hong Wei, Dongni Xia, Li Li, Linpan Liang, Lijun Ning, Cuiliu Gan, Ying Wu

**Affiliations:** ^1^Liuzhou Key laboratory of Infection Disease and Immunology, Research Center of Medical Sciences, Liuzhou People's Hospital Affiliated to Guangxi Medical University, Liuzhou, Guangxi, China; ^2^School of Basic Medical Sciences, Guangxi Medical University, Nanning, Guangxi, China

**Keywords:** baicalin, glycolysis, neutrophil extracellular traps, neutrophils, type I interferon

## Abstract

Type I interferon (IFN-I), a pivotal component of the host's innate antiviral immune system, can induce the formation of neutrophil extracellular traps (NETs) and facilitate inflammatory responses. Baicalin exhibits a range of pharmacological activities, including anti-inflammatory and immunomodulatory effects. It has been reported that neutrophil glycolysis plays a pivotal role in the formation of NETs and the regulation of inflammatory response in immune modulation, regulated by IFN-I. However, it remains unclear whether baicalin regulates IFN-I-induced NETs formation through glycolysis. In this study, we induced the formation of NETs *in vitro* using IFN-I and observed that baicalin significantly reduced the formation of IFN-I-induced NETs. Furthermore, baicalin inhibited the production of pro-inflammatory cytokines, specifically interleukin-1 beta (IL-1*β*) and interleukin-6 (IL-6), as well as the generation of reactive oxygen species (ROS) and chemotactic responses. Our findings further indicated that baicalin could inhibit both lactic acid and ATP levels in IFN-I-induced neutrophils, as well as the expression of glycolytic-related proteins, including HK2, HK3, PKM2, and LDHA. Moreover, following the administration of glycolytic agonists insulin, it was observed that heightened glycolytic activity significantly augmented NETs formation and the release of inflammatory cytokines, potentially regulated by PKC/Raf/MEK/ERK and PI3K/AKT signaling pathways. In conclusion, our findings indicated that baicalin may exert inhibitory effects on IFN-I-induced NETs formation and inflammatory cytokine production by modulating glycolysis, thereby providing further evidence for the potential clinical application of baicalin in the treatment of IFN-I-related inflammatory diseases.

## 1. Introduction

Neutrophils are the primary cell type of the host innate immune system and play a key role in innate immune defense as well as in the pathophysiology of various inflammatory diseases, primarily through the release of neutrophil extracellular traps (NETs) [1]. The main components of NETs, including DNA, histone, neutrophil elastase (NE), myeloperoxidase (MPO), and antimicrobial peptide interleukin-37 (IL-37), could capture and kill pathogenic microorganisms [2]. Several studies have investigated the potential mechanisms underlying the formation of NETs. It has been demonstrated that activation of the Raf-MEK-ERK signaling pathway through protein kinase C (PKC) [3], hyper-citrullination of histones [4–7], and generation of reactive oxygen species (ROS) [8] were indispensable for this process to occur. Although the formation of NETs (NETosis) is beneficial for immune defense, persistent NETosis increases oxidative stress and inflammatory responses. Paul Kubes et al. demonstrated that upon stimulation of neutrophils, PMA activates the PKC and Raf-MEK-ERK signaling pathways. This activation subsequently led to the induction of protein arginine deiminase 4 (PAD4), which catalyzes histone citrullination, resulting in chromatin decondensation, NET formation, and ROS production [3]. Leandro Borges [9] summarized an increase in lung inflammation and NETs from COVID-19. In addition, Han et al. [10] found that in a mouse asthma model with neutrophil-dominated airway inflammation, the formation of NETs exacerbated airway inflammation in mice. Rapid and effective suppression of NETs is the key to preventing excessive immune damage.

Type I interferon (IFN-I, including IFN-*α* and IFN-*β*), as an important regulatory factor in the innate immune response, is the main host defense against viruses and other pathogenic microorganisms and can promote antiviral and immune responses [11, 12]. After secreted from infected cells, IFN-*α*/*β* binds to the IFN-I receptor (IFNAR) and activates the antiviral IFN-stimulating gene (ISG) to regulate innate and adaptive immunity [13]. IFN-I has been reported to have a “double-edged sword” effect. While IFN-I plays a role in antiviral activity, it could also promote the occurrence of inflammatory reactions, leading to immune damage. Krystelle Nganou-Makamdop found that blocking IFN-I signaling during chronic simian immunodeficiency virus (SIV) infection could inhibit IFN-I-associated inflammatory pathways without increasing viral replication [14]. Mature neutrophils express IFNAR and are important target cells for IFN-I. Excessive stimulation of IFN-I could lead to enhanced NETosis in neutrophils, playing a role in immune defense, while the accompanying excessive production of ROS can lead to local inflammation [15, 16]. It has been reported that IFN-I-induced NETs increased lung inflammation in tuberculosis-prone mice [17]. In the study of primary Sjogren's syndrome, it was found that the activation of IFN-I led to mitochondrial damage in neutrophils and the generation of related ROS, ultimately resulting in the production of NETs, which aggravated the severity of the disease [15]. In addition, IFN-I-induced NETs also contribute to the pathogenesis of various inflammatory diseases, such as rheumatoid arthritis (RA), systemic lupus erythematosus (SLE), and acute respiratory distress syndrome (ARDS) [15, 18]. Therefore, the effective regulation of the excessive activation of NETs by IFN-I is beneficial for mitigating immune damage.

Glycolysis is an important driving factor for the recognition of pathogens by innate immune cells. The biological processes associated with neutrophil response are closely related to cellular metabolism [19]. Previous studies have shown that glycolysis appears to be the primary pathway involved in ATP production, phagocytosis, and NETs [20, 21] and that the low abundance of mitochondria in neutrophils has led to the assumption that the biological function of neutrophils is entirely dependent on glycolysis [22]. Similarly, metabolic reprogramming of neutrophils is induced by inflammatory mediators, pathogens, and soluble factors released in a variety of pathologies. In fact, metabolic changes could greatly affect the response of neutrophils to proinflammatory factors or antigens such as viruses and bacteria, leading to the formation of NETs [19]. Previous studies have shown that inhibiting the expression of lactate dehydrogenase A (LDHA) by regulating the PI3K/Akt-HIF-1*α* pathway could suppress glycolysis and contribute to immune suppression of neutrophils during sepsis [23].

Type I interferon (IFN-I) is a key cytokine in immune response, and it has been found that IFN-I could promote the level of cellular glycolysis. Chan et al. [24] found that IFN-I subtype IFN*β* promoted adipocyte glycolysis and amplifies intracellular inflammation. IFN-I is capable of activating multiple signaling pathways, including the phosphatidylinositol 3-kinase (PI3K)/protein kinase B (AKT) pathway, the hypoxia-inducible factor 1-alpha (HIF-1*α*) pathway, and the Janus kinase (JAK)/signal transducer and activator of transcription (STAT) pathway [25]. Among these, the PI3K/Akt and HIF-1*α* pathways were considered to be key regulators of glycolysis. In a mouse model of sepsis, inhibition of LDHA expression by regulating PI3K/Akt-HIF-1*α* pathway could inhibit glycolysis and contribute to immunosuppression of neutrophils [23]. This indicated that the regulation of IFN-I on neutrophil immune defense may be related to the regulation of glycolysis. However, the exact mechanism is still unclear.

Natural products with anti-inflammatory and immunomodulatory functions have a broad application prospect in immunoregulation. Baicalin is an effective flavonoid active ingredient extracted from *Scutellaria baicalin*, which has anti-inflammatory, antibacterial, and immune-enhancing effects [26]. Previous studies have found that baicalin has the effects of anti-inflammatory, inhibition of NETs and regulation of cell metabolism level. In a murine model of polymicrobial sepsis, baicalin administration has been shown to mitigate the elevation of neutrophil counts in peritoneal lavage fluid, reduce the concentrations of proinflammatory cytokines IL-6, TNF-*α*, and chemokine interleukin-17A (IL-17A) in both plasma and peritoneal lavage fluid, and enhance the secretion of the anti-inflammatory cytokine interleukin-10 (IL-10). These effects collectively contribute to improved bacterial clearance [27]. Additionally, baicalin reduces organ damage associated with neutrophil infiltration in the lungs and liver, thereby improving the survival rate of septic mice [27]. Moreover, Li et al. [28] confirmed that baicalin combined with interleukin-8 (IL-8) injected into rat skin could significantly inhibit IL-8-induced neutrophil infiltration. Our previous findings indicated that baicalin exerted a bidirectional modulation of IFNs production and effects, whereby it enhances antiviral IFNs production while concurrently attenuating inflammation and NETs formation of mice neutrophil induced by IFN-I. However, the underlying mechanism remains elusive [29]. Moreover, Shen et al. [30] found that baicalin could reduce the production of chemotactic peptides (fMLP) or PMA-induced ROS in neutrophils and inhibit the activity of MPO, a key component of NETs. The regulation of baicalin on glucose metabolism has also been reported. Huang et al. [31] found that baicalin suppressed the expression of glycolytic enzymes hexokinase 2 (HK2), phosphofructokinase 1 (PFK1), and pyruvate kinase muscle 2 (PKM2) to inhibit glucose metabolism in tumor cells, thereby impeding the growth and function of melanoma tumors. Moreover, baicalin could inhibit blood glucose metabolism in mice and stabilize blood glucose content after high-fat diet [32]. However, there is a paucity of literature addressing the regulatory effects of baicalin on glycolysis, and the precise underlying mechanisms remain inadequately elucidated.

In this study, we elucidated the regulatory mechanism of baicalin on IFN-I-induced neutrophil NETosis and explore whether its potential effects are associated with the modulation of glycolysis. Our aim was to provide a foundational basis for the development and clinical application of baicalin.

## 2. Materials and Methods

### 2.1. Reagents and Antibodies

The sources of reagents and antibodies were described in the Supporting Information Table S1. Baicalin (B20570) was purchased from Shanghai Yuanye Biotechnology Co. Ltd. (Shanghai, China).

### 2.2. Isolation and Identification of Neutrophils

Human peripheral blood in this study was collected from the remaining physical examinational samples of healthy volunteers in Liuzhou People's Hospital. The study was approved by the Medical Ethics Committee of Liuzhou People's Hospital, and written informed consent was obtained from all healthy volunteers. The whole blood was layered on a 2-step Percoll (Sigma Aldrich, USA) gradient (75% and 60% plus cells) and centrifuged with 800×*g* for 30 min at room temperature (RT). Neutrophils were collected from the layer of 60% and 75% Percoll and washed with PBS. Following incubation with red blood cell lysis buffer (Solarbio, China) for 5–10 min, residual erythrocytes were removed. After washing with PBS, neutrophils (1 × 10^6^ cells/mL) were suspended in RPMI 1640 medium containing 10% FBS. Neutrophil viability was established with trypan blue (Solarbio, China) staining. Furthermore, neutrophils suspensions were sequentially incubated with APC-Cy7-conjugated anti-CD45, PE-conjugated anti-CD11b, and FITC-conjugated anti-CD15 antibodies. The incubation was conducted for 20 min under dark conditions. After incubation, the cells were washed with PBS, and the purity of the neutrophil population was evaluated by flow cytometry (Biolegend, USA) (purity ≥ 95%). Considering that neutrophils are suspension cells, the extracted cells were seeded into a multi-well plate at a density of 1 × 10^6^ cells/mL. Subsequently, the cells were co-cultured with IFN (160 ng/mL) and baicalin (5, 10, 20 μg/mL) for 4 or 8 h. After the incubation period, the treated neutrophils were harvested for further detection experiments.

### 2.3. RNA Sequencing (RNA-Seq)

The RNA was extracted from the cells by using the TriQuick Reagent, and then RNA-seq was conducted by Gene Denovo Biotechnology (Guangzhou, China). The RNA-Seq libraries were constructed using the NEBNext Ultra RNA Library Prep Kit for Illumina (NEB#7530, New England Biolabs, Ipswich, MA, USA). Enriched libraries were purified with AMPure XP beads (1.0X, Beckman. Coulter, Brea, CA, USA), and the resulting cDNA. The library was sequenced on the Illumina NovaSeq 6000 platform.

### 2.4. Quantitative Real-Time PCR (qRT-PCR)

The total RNA of neutrophils was isolated by using TriQuick Reagent (Thermo Fisher Scientifc, USA). The commercial cDNA Synthesis Kit (Thermo Fisher Scientifc, USA) was used to reverse-transcribed the RNA into the first-strand cDNA. The expression levels of mRNAs were measured using qPCR, and GAPDH was the internal control. The mRNA expression levels for each gene were measured using the 2^−ΔΔCT^ method. Supporting Information Table S2 shows the primer sequences used for quantitative real-time polymerase chain reaction (qRT-PCR) analyses.

### 2.5. Extraction and Quantification of Cf-DNA

The cf-DNA of treated neutrophil supernatant was isolated and extracted with reagents in the Free DNA Extraction Kit (Uelandy, China) according to the recommended protocol. The concentration of cf-DNA was determined by measuring the absorbance of 1 μL final eluent at 260 nm with a Multiskan SkyHigh Microplate reader (Thermo Scientific, USA).

### 2.6. Measurement of Lactate and ATP

The intracellular lactate content was measured using a Lactic Acid assay kit (Nanjing jiancheng Bioengineering Institute, China) according to the manufacturer's instruction. Briefly, neutrophils were collected after an 8 h treatment with or without IFN-I (160 ng/mL) and baicalin (5, 10, and 20 μg/mL). Removed the medium and washed the cells with PBS. Protein precipitant was added, and cells were homogenized on ice. And then, the homogenates were centrifuged at 4000 rpm for 10 min at 4°C. The supernatant was used for the lactate measurement. The treated cells were lysed and centrifuged at 12,000 rpm for 5 min at 4°C. The ATP content in the supernatant was detected using the ATP assay kit (Beyotime Biotechnology, China) on a Varioskan LUX multimode microplate reader (Thermo Scientific, USA).

### 2.7. Western Blot Analysis

Proteins were extracted from cultured neutrophils using a cell lysis buffer composed of 1× RIPA Lysis Buffer (Shanghai Epizyme Biomedical Technology Co., Ltd, China) and 1× Halt Protease and Phosphatase Inhibitor Cocktail (Thermo, US) (in a ratio of 100 : 1). Protein concentration was detected by BCA Protein Assay Kit (Solarbio, China). After protein quantification, the same amount of protein was separated by SDS-PAGE and transferred to PVDF membranes. The membranes were blocked with 5% fetal bovine serumblocking solution at RT for 1.5 h. And then the membranes were washed three times with 1× TBST (Solarbio, China), followed by incubation at 4°C overnight with antibodies against CiTH3 (Abcam, UK, 1 : 2000), NE (Abcam, UK, 1 : 1000), MPO (Abcam, UK, 1 : 2000), or GAPDH (Abcam, UK, 1 : 4000) and so on. Following this, washed the membranes three times with 1× TBST and incubated with an appropriate horseradish peroxidase (HRP)-coupled secondary antibodies (Abcam, UK, 1 : 4000) at RT for 1 h. Finally, the blots were visualized by enhanced chemiluminescence (ECL) (Bio-rad, USA), and the band intensities were semiquantitatively analyzed using Image J software.

### 2.8. Immunofluorescence Assay

Neutrophils were seeded on sterile coverslips in polylysine-coated 24-well platesat a density of 2 × 10^4^ cells/well, then directly co-cultured with IFN (160 ng/mL) and baicalin (20 μg/mL) for 8 h. The cells were fixed with 4% paraformaldehyde solution at RT for 20 min. After washing the cell with PBS, they were blocked with 5% bovine serum albumin (Beyotime Biotechnology, China) at RT for 1 h, followed by incubating with antibodies against MPO (1 : 100) (Abcam, UK), CiTH3 (1 : 1000) (Abcam, UK), and NE (1 : 200) (Abcam, UK) at 4 °C overnight. After PBS washing, the cells were incubated with Goat anti-Rabbit IgG (H + L) highly Cross-Adsorbed Secondary Antibody (Alexa Fluor Plus 488) (Invitrogen, USA), Donkey anti-Goat IgG (H + L) highly Cross-Adsorbed Secondary Antibody (Alexa Fluor Plus 555) (Invitrogen, USA), and Goat anti-Mouse IgG (H + L) highly Cross-Adsorbed Secondary Antibody (Alexa Fluor 647) (Invitrogen, USA)at RT for 1 h, it was then incubated in 4,6-diamidino-2-phenylindole (DAPI) (Invitrogen, USA, 1 : 500) for 15 min. The cell climbing slices were extensively washed with PBS and covered with Antifade Mounting Medium (Beyotime Biotechnology, China). Stripping (Solarbio, China) was used to remove all antibodies without affecting the antigen bound to the membrane for proteins with the same molecular weight in the same membrane. Then we incubated the second antibody and carried out the same experiment as above. Images were captured by laser confocal microscopy (Leica, Germany), and Image J software was used to evaluate the immunofluorescence intensity of images.

### 2.9. Determination of ROS in Neutrophils

The ROS levels in the treated neutrophils were determined by an oxidation-sensitive fluorescent probe, 2,7-dichlorofuorescein diacetate (DCFH-DA). Intracellular esterase can hydrolyze the probe to nonfluorescent DCFH, and intracellular ROS can further oxidize DCFH into highly fluorescent DCF, which can indirectly reflect ROS levels. DCFH-DA was diluted to 1 µM and incubated with the treated cells in the dark at 37°C for 30 min. And then washed the cells three times with PBS. Peroxidation levels were detected using a BD LSRFortessa flow cytometer (BD, State of New Jersey, USA) or investigated and photographed using a fluorescence microscope and quantified using Image J software.

### 2.10. Neutrophil Chemotaxis Assay

Neutrophils were inoculated in the upper chambers of the 24-well Transwell permeable chambers (3.0 μm) at a density of 2 × 10^4^ cells/well, and additives containing IFN-*α*2 (160 ng/mL) with or without baicalin (5, 10, and 20 μg/mL) were added to the lower chamber of the Transwell, and then incubated at 37°C for 4 or 8 h. All liquid present from the lower chamber of the Transwell was collected and centrifugated. After resuspending with an equal volume solution, cells were treated with Ly6G-FITC and CD11b-PE, and suspension was shielded from light and incubated at 4°C for 30 min. Finally, the number of the cells that migrated to the lower well after either 4 or 8 h incubation was counted using Transwell assay.

### 2.11. Neutrophil Phagocytosis Assay

Neutrophils were inoculated in 6-well plates and cultured them with carboxylate-modified polystyrene Latex beads (Sigma, USA) at 37°C for 1 h, then stop phagocytosis with PBS. Following by resuspending cell with 400 µL pre-cooled PBS, detecting neutrophil phagocytosis by Flow cytometry.

### 2.12. Assessment of NET Formation

Neutrophils (2 × 10^4^ cells/well) were aliquoted into 96-well plates and left to settle for 30 min at 37°C. Plates were incubated for 8 h, after which Syotx Green (final concentration of 6 μM), a cell-impermeable nucleic acid stain with excitation/emission maxima at 504/523 nm, was added. NET formation was subsequently assessed by measuring the mean fluorescence intensity in 96-well plates using a Varioskan LUX Microplate Reader (thermo scientific, USA). Results were assessed by quantifying the mean fluorescence intensity in 96-well plates following background fluorescence subtraction. Additionally, cellular morphology was examined *via* fluorescent microscopy using a fluorescence microscope (Nikon, Japan).

### 2.13. Statistical Analysis

The data were analyzed by using SPSS version 26 (IBM, Chicago, USA) by one-way analysis of variance (ANOVA) followed by an LSD for the post hoc test, and graphical representations were generated using Prism GraphPad version 8.0.1 (GraphPad Software). The results were expressed as mean ± standard deviation (SD). The data shown were representative of results from three independent experiments performed in duplicates unless otherwise indicated. A *p* value of < 0.05 was considered significant.

## 3. Results

### 3.1. Baicalin Inhibited the Formation of NETosis Mediated by Type I IFN

**Figure 1:**
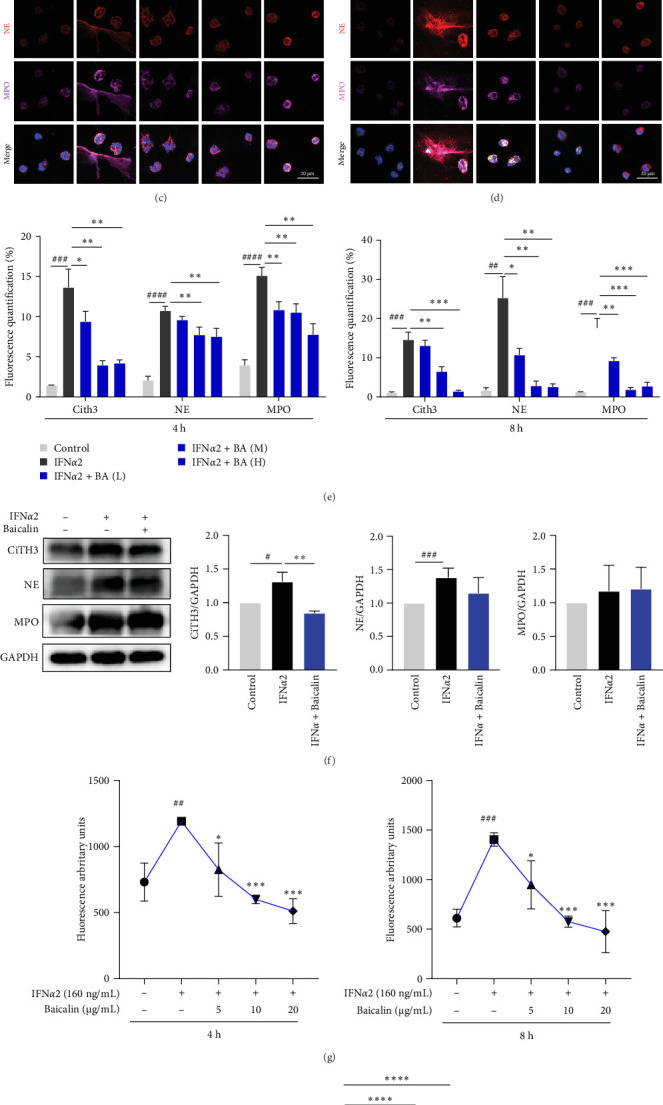
Baicalin suppressed the production of NETs in IFN*α*2-induced neutrophil. (A) Heatmap showing the NETs-associated genes that were significantly differentially expressed between control and IFN*α*2 groups. (B) The Kyoto Encyclopedia of Genes and Genomes (KEGG) pathways enriched with differentially expressed genes between control and IFN*α*2 groups. (C–E) Neutrophils were stimulated with IFNα2 for 4 h (C) or 8 h (D), with or without Baicalin administration. Representative confocal microscopy images of NETs markers (CitH3, green; NE, red; MPO, purple) and relative quantitative analysis of fluorescence were detected (E). Scale bars, 20 μm. (F) Neutrophils were stimulated with IFN*α*2 for 8 h, with or without baicalin administration. Cell lysates were analyzed by Western blotting for NETs-associated proteins. (G) The fluorescence of NETs stained with cell-impermeable Sytox Green was measured using an excitation wavelength of 504 nm and an emission wavelength of 523 nm. Background fluorescence was subtracted, and the abundance of NETs was expressed in arbitrary fluorescence units. (H) Cell supernatants were analyzed for the secretion levels of cf-DNA. Data are shown as mean ± SD, *⁣*^#^*p* < 0.05, *⁣*^##^*p* < 0.01, *⁣*^###^*p* < 0.001, *⁣*^####^*p* < 0.0001 versus Control group; *⁣*^*∗*^*p* < 0.05, *⁣*^*∗∗*^*p* < 0.01, *⁣*^*∗∗∗*^*p* < 0.001, *⁣*^*∗∗∗∗*^*p* < 0.0001 versus IFN*α*2 group. *Note:* BA (L), BA (M), and BA (H)represented baicalin concentrations of 5, 10, and 20 μg/mL, respectively. NETs, neutrophil extracellular traps; SD, standard deviation.

We first conducted transcriptomic analysis by RNA-seq and found that (Figure 1A,B), compared with the normal group, NET-related genes (H3-3B, H4C14, H4C8, GSDMD, H4C15, CASP1, and CASP4) in the IFN*α*2 group showed significant changes, showing significant upregulation. Further analysis using the KEGG pathways revealed that differentially expressed genes were enriched in NETs signaling pathway, chemokine pathway, and inflammation-related signaling pathway. Then, by confocal immunofluorescence assay (Figure 1), we found that compared with the normal group, IFN*α*2-stimulated neutrophils for 4 and 8 h could significantly induce increased expression of NETs-related proteins CiTH3, NE and MPO, and an obvious reticular structure was observed, while baicalin significantly inhibited this phenomenon. Incubation of neutrophils with IFN*α*2 induced significant morphological changes at 4 and 8 h post-stimulation, leading to the formation of NETs that stained positively with Sytox Green, a dye impermeable to cells with intact membranes. Following baicalin treatment, the formation of NETs was markedly inhibited (Figure 1G and Supporting Information Figure S2). Cells stimulated with IFN*α*2 displayed characteristic diffuse NET morphology (Supporting Information Figure S2). Total fluorescence levels, measured using Sytox Green, were used to quantify NETs formation, and the results correlated well with the fluorescence imaging data (Figure 1G). In addition, we further found through Western bloting experiments that IFN*α*2 stimulated neutrophils to increase the expression of CiTH3 and NE proteins, while baicalin could significantly inhibit the expression of CiTH3 (Figure 1F). When stimulated by infection or inflammation, neutrophils release cf-DNA (circulating free DNA) as a marker of inflammatory response. We found that IFN*α*2 could induce neutrophils to produce more cf-DNA, while baicalin could significantly inhibit its production (Figure 1H). These results suggested that baicalin could inhibit the development of IFN*α*2-stimulated NETs.

### 3.2. Baicalin Decreased the Transcription Levels of IFN-I-Stimulated Neutrophils Inflammatory Cytokines and ROS Production Levels

**Figure 2:**
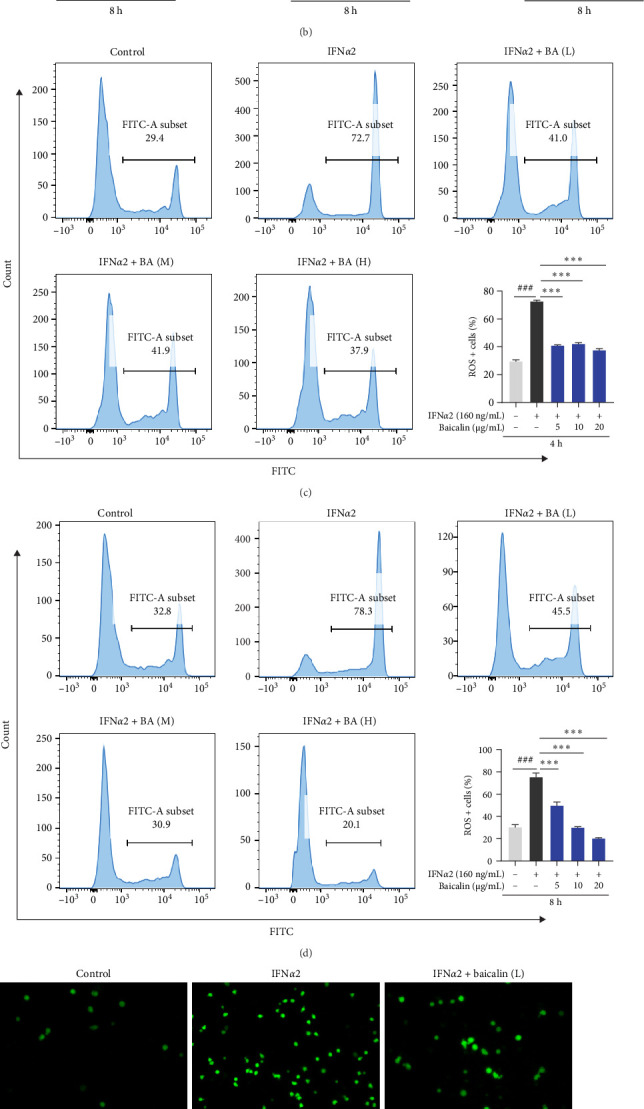
Baicalin diminished the transcription levels of proinflammatory cytokines and the production levels of ROS in Neutrophils stimulated by IFN-I. IFN*α*2-induced neutrophils were treated with baicalin at concentrations of 5, 10, and 20 μg/mL for 4 h (A, C, and E) and 8 h (B, D, and F). (A and B) The mRNA levels of *TNF-α*, *IL-1β*, *IL-6*, *CCL2*, *CXCL10*, and *IL-10* were detected by qRT-PCR. (C–F) The levels of ROS production were measured by flow cytometry (C and D) and fluorescence imaging (E and F) using a DCFH-DA probe. Scale bars, 400 μm. Data are shown as mean ± SD, *⁣*^#^*p* < 0.05, *⁣*^##^*p* < 0.01, *⁣*^###^*p* < 0.001 versus Control group; *⁣*^*∗*^*p* < 0.05, *⁣*^*∗∗*^*p* < 0.01, *⁣*^*∗∗∗*^*p* < 0.001 versus IFN*α*2 group. DCFH-DA, 2,7-dichlorofuorescein diacetate; ROS, reactive oxygen species; qRT-PCR, quantitative real-time polymerase chain reaction.

Neutrophils activation leads to the development of NETs, accompanied by the production of inflammatory cytokines [33]. To investigate the effects of IFN*α*2 on inflammatory cytokines in neutrophils and the effect of baicalin treatment, the transcription levels of *TNF-α*, *IL-1β*, *IL-6*, *CCL2*, *CXCL10*, and *IL-10* were measured. As shown in Figure 2A, B, IFN*α*2 stimulated neutrophils for 4 and 8 h, respectively, and the transcription levels of inflammatory cytokines *TNF-α*, *IL-1β*, *IL-6*, *CCL2*, and *CXCL10* were significantly increased compared with the normal group. After baicalin intervention for 4 h, the transcription levels of inflammatory cytokines *IL-6*, *CCL2*, and *CXCL10* were decreased, mainly in the high-concentration baicalin group. We also observed an increase in the mRNA level of the anti-inflammatory cytokine *IL-10* after the intervention of baicalin. However, after 8 h of baicalin treatment, the transcription levels of inflammatory cytokines *IL-1β*, *IL-6*, and *CXCL10* were decreased, especially *IL-6* and *CXCL10*, and the transcription levels of *IL-10* were also increased. However, a low concentration of baicalin promoted the transcription levels of inflammatory cytokines such as *TNF-α*, *IL-1β*, and *IL-6* during the early stage (4 h), suggesting that baicalin exhibited a dual effect on cytokine production at low concentrations during this period. The precise dynamic regulatory mechanism required further investigation. When neutrophils capture pathogens and play an immune defense role, they are accompanied by the production of ROS, which promotes the development of inflammation [34, 35]. What is more, the production of ROS may also be a mechanism for the formation of NETs [36]. Therefore, the elimination of ROS during the immune defense mediated by neutrophils could reduce the immune damage caused by the defense process. Through flow cytometry (Figure 2C,D), we found that IFN*α*2 stimulation induced a significant increase in the production levels of ROS in neutrophils, which could be effectively suppressed by baicalin treatment. These findings were consistent with those obtained by fluorescence imaging ROS using DCFH-DA probes (Figure 2E,F).

### 3.3. The Phagocytosis of Neutrophils Induced by IFN-I Was Inhibited by Baicalin, While Its Chemotaxis Was Increased in the Early Stage (4 h) and Had No Effect on Chemotaxis in the Late Stage (8 h)

**Figure 3:**
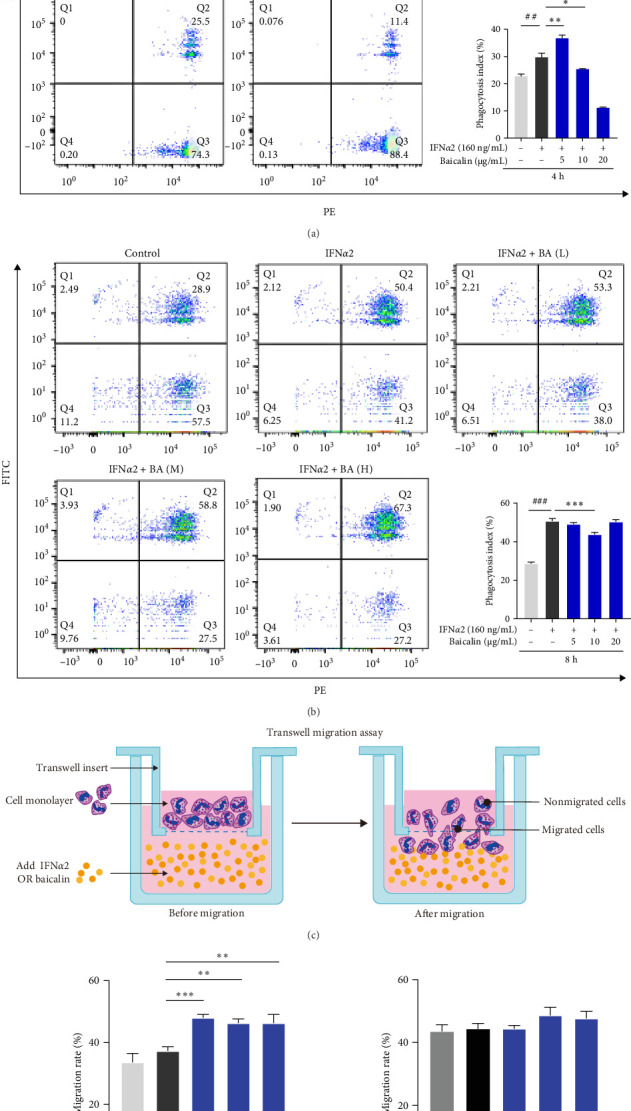
Baicalin exhibited inhibitory effects on IFN-I-induced neutrophil phagocytosis, while promoting its chemotactic function. (A–D) Neutrophils were stimulated by IFN*α*2, with or without Baicalin treatment for 4 and 8 h, respectively. Flow cytometric analysis of neutrophil phagocytic index (phagocytosis of latex beads) (A and B). Schematic diagram of Transwell migration assay (C). Transwell experimental analysis of neutrophil chemotaxis (D). Data are shown as mean ± SD, *⁣*^##^*p* < 0.01, *⁣*^###^*p* < 0.001 versus Control group (negative control); *⁣*^*∗*^*p* < 0.05, *⁣*^*∗∗*^*p* < 0.01, *⁣*^*∗∗∗*^*p* < 0.001 versus IFN*α*2 group.

Phagocytosis is a process involving neutrophil binding and internalization of pathogens [37]. We studied the effect of baicalin on IFN*α*2-stimulated neutrophil phagocytosis by flow cytometry. The results showed that (Figure 3A,B), compared with the normal group, IFN*α*2 could promote the phagocytosis of neutrophils after 4 and 8 h stimulation, and the intervention of baicalin inhibited the phagocytosis of neutrophils, mainly in low and high concentrations of baicalin. In addition, neutrophils have a chemotactic function, and because of their chemotactic nature, neutrophils quickly migrate to the site of infection after recognizing the invader [38]. A Transwell migration assay is widely utilized to investigate the chemotaxis of neutrophils (Figure 3C). The results demonstrated that IFN*α*2 stimulation had no effect on neutrophil chemotactic function, while baicalin could increased neutrophil chemotactic level in the early stage (4 h). However, the late stage (8 h) had no effect on the chemotactic function of neutrophils (Figure 3D). The findings suggested that IFN-I promoted neutrophil phagocytosis without affecting the chemotactic function, while baicalin inhibited the IFN*α*2-induced phagocytosis of neutrophils and promoted the chemotactic response of neutrophils, which was conducive to the aggregation of neutrophils to the site of infection or injury and played its immune defense functions. Furthermore, based on the findings presented in Figure 2A,B, a low concentration of baicalin promoted the production of inflammatory cytokines in neutrophils during the early stage (4 h), whereas a high concentration of baicalin inhibited cytokine production. At the later stage (8 h), baicalin consistently exhibited an inhibitory effect on the production of inflammatory cytokines. This indicated that baicalin exerted a dual effect on the immune response of neutrophils, enhancing their immune function in the early phase while inhibiting excessive immune responses in the later stages.

### 3.4. Baicalin May Regulate IFN-I-Mediated Neutrophils NETosis Through PKC/Raf/MEK/ERK and PI3K/AKT Pathways

**Figure 4:**
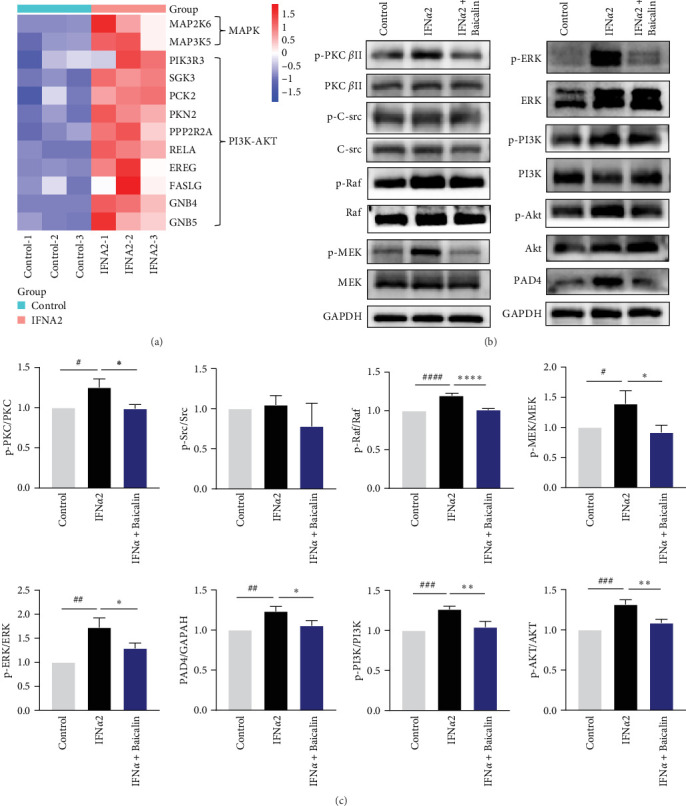
Baicalin may suppressed IFN-I-mediated NETosis by inhabiting PKC, Raf, MEK, ERK, PI3K, and AKT phosphorylation in neutrophils. (A) Heatmap showing the MAPK and PI3K-AKT signaling pathway-related genes that were significantly differentially expressed between control and IFN*α*2 groups. (B and C) Neutrophils were stimulated by IFN*α*2, with or without baicalin (20 μg/mL) treatment for 8 h. Subsequently, the proteins expression levels of PKC, Raf, MEK, ERK, PI3K, and AKT were detected using Western blotting. Data are shown as mean ± SD, *⁣*^##^*p* < 0.01, *⁣*^###^*p* < 0.001 versus Control group (negative control); *⁣*^*∗*^*p* < 0.05, *⁣*^*∗∗*^*p* < 0.01 versus IFN*α*2 group.

Activation of PKC and Raf-MEK-ERK signaling pathways has been reported to influence the formation of NETs in PMA-stimulated neutrophils [39]. In addition, it has been found that the activation of PI3K and Src kinases could also induce the formation of NETs, and PI3K could be activated by it as the downstream of IFN-I [40, 41]. Next, we would explore the pharmacological mechanism by which baicalin regulated IFN-I-induced NETs formation and inflammatory cytokine transcription. Initially, we conducted transcriptomic analysis of IFN-I-stimulated neutrophils and observed that IFN-I augmented the expression of genes associated with MAPK and PI3K/AKT signaling pathways in neutrophils (Figure 4A). Furthermore, as depicted in Figure 4B,C, IFN*α*2 stimulation elicited a notable augmentation in the phosphorylation levels of PKC *β*Ⅱ, ERK, PI3K, and AKT within neutrophils. Conversely, baicalin treatment exhibited a pronounced reduction in the phosphorylation levels of these proteins. In addition, we observed phosphorylation of PAD4 protein associated with NETs formation in neutrophils under IFN-I induction, which was inhibited by baicalin treatment.

We further activated the ERK signaling pathway using an ERK agonist (C16-PAF) to verify the role of the ERK signaling pathway in baicalin's inhibition of IFN-I-induced NETs levels. As illustrated in Supporting Information Figure S3A,B, C16-PAF treatment significantly enhanced ERK phosphorylation and the expression of NETs-related proteins, specifically CiTH3 and MPO. Additionally, baicalin administration effectively attenuated C16-PAF-induced ERK phosphorylation and CiTH3 expression in neutrophils, as well as reduced the transcription levels of inflammatory cytokines TNF-*α* and IL-1*β* (Supporting Information Figure S3D). Furthermore, the levels of NETs were quantified using Sytox Green. The results revealed that the fluorescence intensity in the C16-PAF group was significantly higher than that in the control group. Treatment with baicalin, however, markedly reduced the C16-PAF-induced NETs formation (Supporting Information Figure S3C). These results suggested that baicalin may inhibit NETosis levels and inflammatory development in neutrophils through PKC/MEK/ERK and PI3K/AKT pathways.

### 3.5. Baicalin Inhibited the Glycolysis Level of Neutrophils Induced by IFN-I

**Figure 5:**
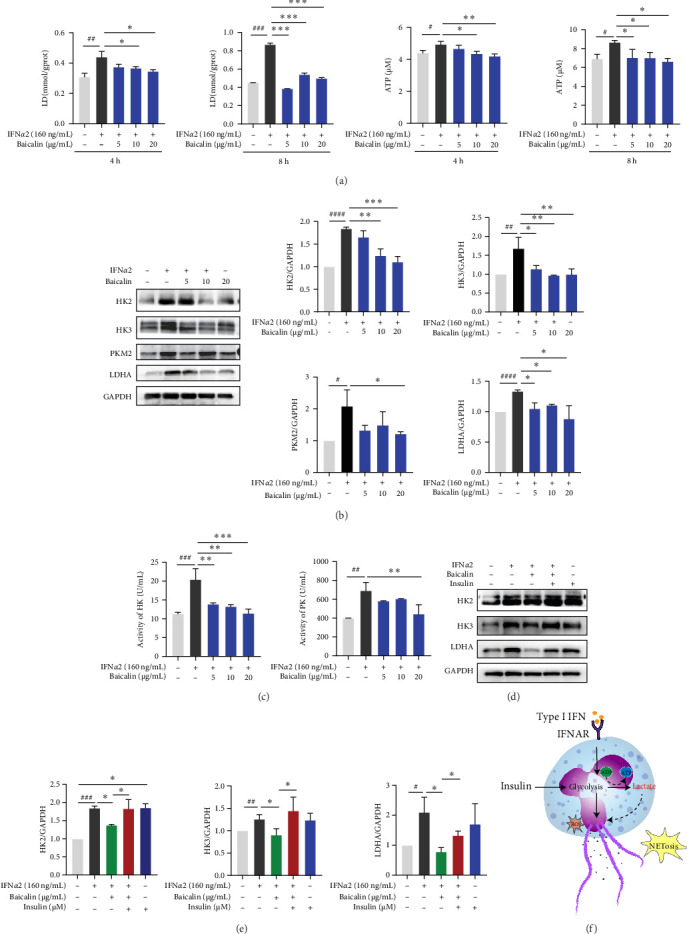
Baicalin suppressed IFN-I-induced glycolysis of neutrophils. (A) The neutrophils were stimulated by IFN*α*2, with or without treatment of baicalin (5, 10, and 20 μg/mL), for 4 and 8 h, respectively. Subsequently, the levels of lactic acid and ATP in neutrophils were measured using Lactic Acid assay kit and ATP assay kit respectively. (B) Neutrophils were stimulated by IFN*α*2, with or without baicalin (5, 10, and 20 μg/mL) treatment for 8 h, and the proteins expression levels of HK2, HK3, PKM2, and LDHA were detected using Western blotting. (C) The enzyme activities of HK and PK were detected by enzyme activity detection kits. (D and E) The neutrophils were treated with the glycolytic agonists insulin for 1 h before IFN*α*2 transfection and Baicalin treatment. The proteins expression levels of HK2, HK3, and LDHA were detected using Western blotting. (F) Schematic diagram of the regulation of neutrophil glycolysis by IFN-I. Data are shown as mean ± SD, *⁣*^#^*p* < 0.05, *⁣*^##^*p* < 0.01, *⁣*^###^*p* < 0.001, *⁣*^####^*p* < 0.0001 versus Control group; *⁣*^*∗*^*p* < 0.05, *⁣*^*∗∗*^*p* < 0.01, *⁣*^*∗∗∗*^*p* < 0.001 versus IFN*α*2 group. LDHA, lactose dehydrogenase A; SD, standard deviation.

When neutrophils migrate to the site of infection and accumulate to perform immune defense functions, they require energy provided by mitochondrial oxidative phosphorylation (OXPHOS), whereas neutrophils are normally heavily dependent on glycolysis [42]. In this study (Figure 5A and Supporting Information Figure S4), we observed that IFN-I stimulation led to an increase in lactate and ATP levels within neutrophils, while baicalin intervention resulted in a decrease of these levels. We further observed that IFN-I upregulated the expression levels of neutrophil glycolytic-related proteins (HK2, HK3, PKM2, and LDHA), whereas baicalin intervention attenuated the expression levels of HK2, HK3, PKM2, and LDHA (Figure 5B). Moreover, using the enzyme activity detection kit, we observed that the activity of hexokinase (HK) and pyruvate kinase (PK) was significantly enhanced upon stimulation with IFN*α*2. However, baicalin exhibited a marked inhibitory effect on the enzymatic activities of HK and PK, particularly at higher concentrations (Figure 5C). We subsequently treated neutrophils with glycolytic agonists (insulin) and observed that the intervention effect of baicalin was attenuated by the agonist, leading to an enhancement in the glycolytic activity of neutrophils (Figure 5D,F). These results indicated that baicalin could inhibit the level of neutrophil glycolysis induced by IFN-I.

### 3.6. Baicalin Attenuated IFN-I-Induced Neutrophil NETosis by Modulating Glycolytic Metabolism, Which May Be Mediated by PKC/Raf/MEK/ERK and PI3K/AKT Signaling Pathways

**Figure 6:**
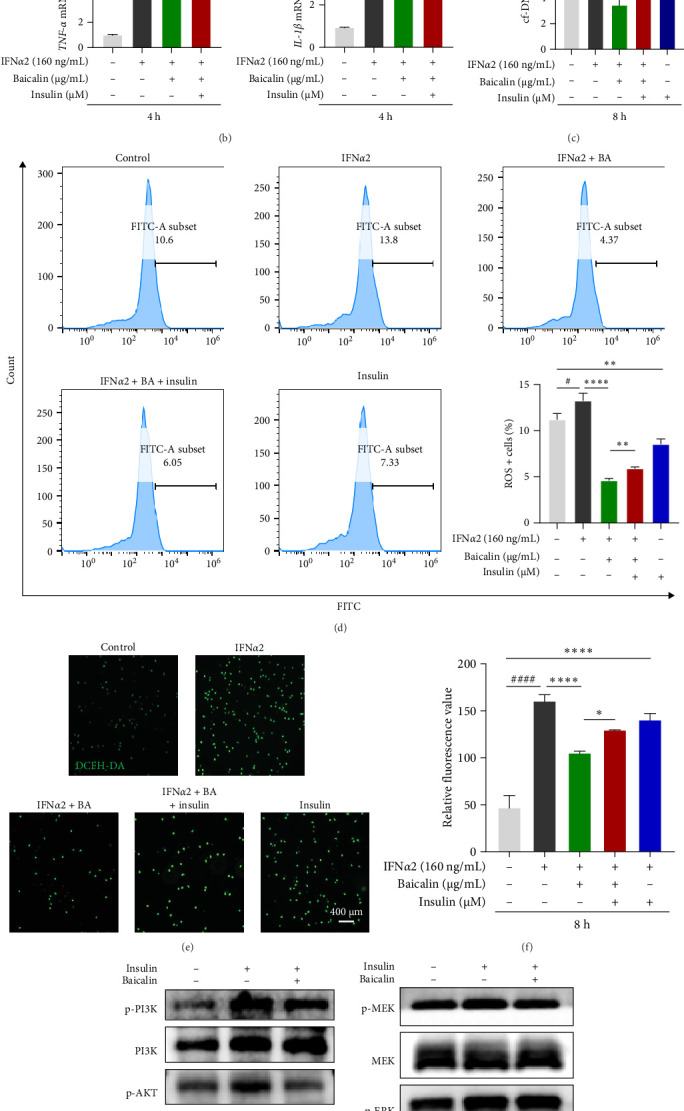
Baicalin regulated glycolysis by inhibiting PKC, Raf, MEK, ERK, PI3K, and AKT phosphorylation, thereby inhibiting IFN-I-induced NETs and ROS levels. The neutrophils were treated with the glycolytic agonists insulin for 1 h before IFN*α*2 transfection and baicalin treatment. (A) Representative confocal microscopy images of NETs markers (CitH3, green; NE, red; MPO, purple) and relative quantitative analysis of fluorescence were detected. Scale bars, 20 μm. (B) The mRNA levels of *TNF-α* and *IL-1β* were detected by qRT-PCR. (C) The secretion level of cf-DNA in the cell supernatant was analyzed. (D–F) The levels of ROS production were assessed by flow cytometry (D) and fluorescence imaging utilizing DCFH-DA fluorescent probes (E–F). Scale bars, 400 μm. (G–H) The proteins expression levels of PKC, Raf, MEK, ERK, PI3K, and AKT were detected using Western blotting. Data are shown as mean ± SD, *⁣*^#^*p* < 0.05, *⁣*^##^*p* < 0.01, *⁣*^###^*p* < 0.001, *⁣*^####^*p* < 0.0001 versus Control group; *⁣*^*∗*^*p* < 0.05, *⁣*^*∗∗*^*p* < 0.01, *⁣*^*∗∗∗*^*p* < 0.001, *⁣*^*∗∗∗∗*^*p* < 0.0001 versus IFN*α*2 group. NETs, neutrophil extracellular traps; qRT-PCR, quantitative real-time polymerase chain reaction; ROS, reactive oxygen species.

It has been reported that the formation of NETs was regulated by glycolysis, and the imbalance of their regulation created a proinflammatory environment and led to reduced neutrophil function [20, 43]. Our findings demonstrated that baicalin effectively suppressed IFN-I-induced NETosis in neutrophils, while the expression of key proteins associated with NET formation, including CiTH3, NE, and MPO, was upregulated in insulin-treated neutrophils (Figure 6A). Additionally, there was an observed increase in the release of cf-DNA (Figure 6C). Moreover, baicalin intervention inhibited the production of inflammatory cytokines *TNF-α*, *IL-1β*, and ROS during the release of NETs induced by IFN-I. However, insulin treatment significantly increased the transcription level of *IL-1β* and expression level of ROS (Figure 6). We conducted further investigation into the pathways associated with glycolysis regulation of NETs, and the findings revealed that insulin stimulation led to an increase in protein phosphorylation levels of PKC, Raf, MEK, ERK, PI3K, CiTH3, and NE (Figure 6G,H). Whereas, the intervention of baicalin was found to attenuate insulin-induced phosphorylation of PKC, Raf, MEK, PI3K, CiTH3, and NE proteins in neutrophils. The findings suggested that the increase of the upregulation of neutrophil glycolysis could enhance the formation of NETs and promote inflammatory response progression, while baicalin could inhibit the level of IFN-I-induced NETs in neutrophils by inhibiting the glycolysis level, which was mainly related to PKC/Raf/MEK/ERK and PI3K/AKT pathways.

## 4. Discussion

In this study, we assessed the impact of baicalin on IFN-I-induced NETs formation and its associated regulatory mechanisms, elucidating the intricate interplay between this process and glycolysis. Neutrophils were hailed as the vanguard against invading pathogens, effectively eliminating them through the release of NETs. Previous studies have linked IFN-I with the facilitation of NETs formation. Neutrophils were the main target cells of IFN-I, playing a crucial role in the initiation and exacerbation of inflammation. Initially, we observed that IFN-I stimulation of neutrophils led to an upregulation in the transcription levels of their factors related to NETs, as determined through transcriptomic analysis. Subsequent in vitro investigations demonstrated that treatment with IFN-I resulted in the upregulation of NETs, accompanied by enhanced generation of ROS and proinflammatory cytokines. The production of ROS is believed to play a crucial role in the progression of inflammatory diseases such as RA [44], sepsis [45, 46], ARDS [47], and ischemia-induced tissue damage [48]. However, baicalin significantly attenuated the levels of NETs and ROS induced by IFN-I, thereby mitigating the immune activation-induced inflammatory response. Furthermore, baicalin demonstrated a sophisticated dual regulatory mechanism on the expression of neutrophilic inflammatory cytokines, indicating its ability to finely tune the immune response of neutrophils. It enhanced immune function during the initial phase while restraining excessive immune reactions in the subsequent phase, thereby maintaining a delicate balance within the immune system. Phagocytosis of neutrophil is a pivotal process in host defense and neutrophil-mediated inflammation, enabling internalization and degradation of granular entities such as viruses, dead cells, and infected cells. Following phagocytosis, neutrophils form phagosomes containing ROS to eliminate substances like viruses [49]. However, our research has revealed that baicalin significantly suppressed IFN-I-induced neutrophil phagocytosis during the early stage (4 h), particularly at high concentrations, while it exhibited no significant inhibitory effect on neutrophils during the late stage (8 h). These findings implied that there is temporal variability in the dynamics of neutrophil immune function, which necessitated further investigation into the underlying reasons and mechanisms. In summary, baicalin effectively attenuated the sustained NETosis induced by IFN-I and mitigates its inflammatory response.

Previous research has demonstrated that the formation of NETs could be influenced by the PKC and Raf/MEK/ERK signaling pathways [39]. Furthermore, the PI3K and AKT pathways play crucial roles in the inflammatory response [50]. Additionally, relevant studies have previously identified PI3K and AKT as pivotal signaling pathways that govern the formation of NETs [51]. The aforementioned signaling pathways, however, are mediated by IFN-I [25]. The *in vitro* studies revealed that IFN-I stimulation of neutrophils was found to induce the activation of PKC/Raf/MEK/ERK and PI3K/AKT pathways, which aligned with the findings from KEGG pathway analysis (Figure 1B) and Gene Ontology (GO) analysis (Supporting Information Figure S1). However, baicalin intervention effectively attenuated the phosphorylation levels of proteins involved in these signaling cascades. These findings suggested that the activation of neutrophil-derived NETs by IFN-I is primarily mediated through the PKC/Raf/MEK/ERK and PI3K/AKT signaling pathways. However, intervention with baicalin significantly attenuated the activation of these pathways, thereby suppressing NETs levels.

There is increasing evidence that glucose metabolic reprogramming plays an important role in the regulation of inflammatory response [52, 53]. The primary metabolic pathway of neutrophils predominantly relies on glycolysis, wherein the conversion of pyruvate to lactic acid is catalyzed by lactate dehydrogenase (LDH). Simultaneously, there is an increase in glucose consumption and lactic acid content, leading to substantial ATP production [54]. In this study, we observed an elevation in lactic acid and ATP levels in neutrophils induced by IFN-I, whereas baicalin effectively attenuated this increase. HK, PK, and LDHA are the key enzymes of glycolysis. Our further investigation has revealed that IFN-I significantly enhanced the expression of HK2, HK3, pyruvate dehydrogenase kinase isozyme 3 (PDK3), M2-type pyruvate kinase (PKM2), and LDHA. Additionally, baicalin effectively suppressed the activity of glycolytic enzymes. These findings suggested that IFN-I promoted neutrophil glycolysis levels, aligning with Chan et al.'s report [24], while baicalin exerted a significant inhibitory effect on this metabolic pathway. Moreover, the glycolytic activity of neutrophils exhibited a significant correlation with the formation of NETs. Notably, studies have demonstrated that the targeted disruption of glycolysis using genetic knockout enzymes and small molecule inhibitors effectively suppressed the formation of NETs [55, 56]. We observed that insulin-induced glycolytic activation of neutrophils resulted in the phosphorylation of PKC, Raf, MEK, ERK, and PI3K, as well as upregulation of NETs expression (CiTH3 and NE). In contrast, baicalin inhibited the phosphorylation of proteins associated with PKC/Raf/MEK/ERK and PI3K/AKT signaling pathways, along with suppressing the expression of IFN-I-induced NETs in neutrophils. Collectively, these findings suggested that baicalin may mitigate the hyperactivation of neutrophils induced by IFN-I through suppression of glycolysis via the PKC/Raf/MEK/ERK and PI3K/AKT signaling pathways. The induction of NETs and inflammation by IFN-I plays a crucial role in the pathogenesis of infectious and autoimmune diseases. Our findings underscored the potential significance of glycolysis in driving IFN-I-mediated neutrophil-associated inflammatory diseases, thereby highlighting the imperative for further investigation into IFN-I-stimulated neutrophils to gain deeper insights into disease mechanisms. Consequently, targeting the IFN-glycolytic-NETs axis in immune cells through novel pharmacological interventions could provide promising therapeutic strategies for ameliorating neutrophil-related inflammatory disorders driven by type I IFN, thereby offering valuable insights into disease mechanisms.

## 5. Conclusion

Our study has provided novel insights into the inhibitory effects of baicalin on IFN-I-induced NETs and excessive inflammation through the regulation of glycolysis, thereby uncovering a previously unknown anti-inflammatory and antioxidant mechanism of baicalin. These findings have presented compelling evidence for the potential clinical application of baicalin in the treatment of inflammatory diseases.

## Data Availability

All the original data of this experiment can be obtained from the corresponding author if necessary.

## Supporting Information

Supporting Information Table S1: Reagents and antibodies. Table S2: Primer sequences for qPCR. Figure S1: The GO pathways enriched with differentially expressed pathways between control and IFN*α*2 groups. Figure S2: Fluorescence images of NETs were quantified using the cell-impermeable nucleic acid stain Sytox Green. Figure S3: Baicalin exhibited inhibitory effects on neutrophil NETs and glycolysis levels induced by C16-PAF. The neutrophils were treated with the ERK agonist C16-PAF for 1 h before Baicalin treatment. (A–B) The proteins expression levels of p-MEK, MEK, p-ERK, ERK, Cith3, MPO, HK2, HK3, and LDHA were detected using Western blotting (A) and relative quantitative analysis (B); (C) the fluorescence of NETs stained with cell-impermeable Sytox Green was measured using an excitation wavelength of 504 nm and an emission wavelength of 523 nm; (D) the mRNA levels of inflammatory cytokines *TNF-α* and *IL-1β* were detected by qRT-PCR. Figure S4: The neutrophils were stimulated by IFN*α*2, with or without treatment of Baicalin (5, 10, 20 μg/mL), for 6 h and 12 h, respectively. Subsequently, the levels of ATP in neutrophils were measured using ATP assay kit respectively. Figure S5: (A–B) Gating strategies in flow cytometry for neutrophil ROS and phagocytosis function analysis.
