# Supplementary material for: Baicalin Modulates Glycolysis via the PKC/Raf/MEK/ERK and PI3K/AKT Signaling Pathways to Attenuate IFN-I-Induced Neutrophil NETosis
Source: Mediators Inflamm. 2025 May 19;2025:8822728. doi: 10.1155/mi/8822728 (PMC12105894; doi:10.1155/mi/8822728)
Supplement: Supporting Information — Table S1: Reagents and antibodies. Table S2: Primer sequences for qPCR. Figure S1: The GO pathways enriched with differentially expressed pathways between control and IFNα2 groups. Figure S2: Fluorescence images of NETs were quantified using the cell-impermeable nucleic acid stain Sytox Green. Figure S3: Baicalin exhibited inhibitory effects on neutrophil NETs and glycolysis levels induced by C16-PAF. The neutrophils were treated with the ERK agonist C16-PAF for 1 h before Baicalin treatment. (A–B) The proteins expression levels of p-MEK, MEK, p-ERK, ERK, Cith3, MPO, HK2, HK3, and LDHA were detected using Western blotting (A) and relative quantitative analysis (B); (C) the fluorescence of NETs stained with cell-impermeable Sytox Green was measured using an excitation wavelength of 504 nm and an emission wavelength of 523 nm; (D) the mRNA levels of inflammatory cytokines TNF-α and IL-1β were detected by qRT-PCR. Figure S4: The neutrophils were stimulated by IFNα2, with or without treatment of Baicalin (5, 10, 20 μg/mL), for 6 h and 12 h, respectively. Subsequently, the levels of ATP in neutrophils were measured using ATP assay kit respectively. Figure S5: (A–B) Gating strategies in flow cytometry for neutrophil ROS and phagocytosis function analysis. [file 8822728.f1.docx]

**Baicalin modulates glycolysis *via* the PKC/Raf/MEK/ERK and PI3K/AKT signaling pathways to attenuate IFN-I-induced neutrophil NETosis**

Hong wei^1^^†^, Dongni Xia ^1,2†^, Li Li^1^, Linpan Liang^1,2^, Lijun Ning^1^, Cuiliu Gan^1^, Ying Wu^1^*

^†^ Hong wei and Dongni Xia equal contributions to this work.

*Corresponding author. Tel.: +86-0772-2663136.

E-mail address: aqiwuying@163.com (Ying Wu).

*^1^Liuzhou Key laboratory of infection disease and immunology, Research Center of Medical Sciences, Liuzhou People's Hospital Affiliated to Guangxi Medical University, Liuzhou, Guangxi, China.*

*^2^School of Basic Medical Sciences, Guangxi Medical University, Nanning, Guangxi, China.*

**Supporting Information**

**Supporting tables**

**Table S1** Reagents and antibodies

| **Reagents** | **Company name (catalog number)** | **City/State, Country** |
| --- | --- | --- |
| IFNα2 | MCE (#HY-12836A) | Shanghai, CN |
| Baicalin | Shanghai Yuanye Biotechnology Co (#B20570) | Shanghai, CN |
| Insulin | MCE (#HY-P0035-10mg) | Shanghai, CN |
| C16-PAF | MCE (#HY-108635-5mg) | Shanghai, CN |
| DAPI | UElandy (#A4084) | Suzhou, CN |
| RPMI 1640 | Thermo Scientifie (#C11875500BT) | Massachusetts.US |
| Penicillin/ streptomycin | Solarbio (#P1400) | Beijing, CN |
| Fetal Bovine Serum, Premium Plus | Thermo Scientific (#A5669701) | Massachusetts, US |
| Fetal Bovine Serum, Prime | ExCell Bio (#FSP500) | Suzhou, CN |
| Cell Counting Kit-8(CCK-8) | BIOSHARP (#BS350B) | Beijing, CN |
| TriOuick Reagent | Solarbio (#R1100) | Beijing, CN |
| SuperSignalim^TM^ West Atto | Thermo Scientific (#A38555) | Massachusetts, US |
| Omni-ECL^TM^ Femto Light Chem iluminescence Kit | EpiZyme (#S0201) | Shanghai, CN |
| Enhanced BCA Protein Assay Kit | Beyotime (#P0009) | Shanghai, CN |
| 4% paraformaldehyde | BIOSHARP (#BL539A) | Beijing, CN |
| Reactive oxygen species assay kit | Solarbio (#CA1410) | Beijing, CN |
| Carboxylate-modified polystyrene latex beads | Sigma-Aldrich (#L4655) | St. Louis, US |
| Free DNA Extraction Kit | Uelandy (#M7417M) | Suzhou, CN |
| Lactic Acid assay kit | Nanjing jiancheng Bioengineering Institute (#A019-2-1) | Nanjing, CN |
| ATP test kit | Beyotime (#S0026) | Shanghai, CN |
| Halt Protease and Phosphatase Inhibitor Cocktail (100X) | Thermo Scientific (#78442) | Massachusetts, US |
| RIPA Lysis Buffer | Shanghai Epizyme Biomedical Technology Co., Ltd（#PC101） | Shanghai, China |
| Antifade Mounting Medium | Solarbio (#S2100) | Beijing, CN |
| Hexokinase (HK) kit | Solarbio (#BC0740) | Beijing, CN |
| Pyruvate kinase (PK) test kit | Solarbio (#BC0540) | Beijing, CN |
| Percoll^TM^ | Cytiva (#17089101) | Uppsala, SE |
| phosphate buffered solution | Solarbio (#P1010-500) | Beijing, CN |
| 10×phosphate buffered solution | Solarbio (#P1025) | Beijing, CN |
| Red Blood Cell Lysis Buffer | Solarbio (#R1010) | Beijing, CN |
| Trypan Blue | Solarbio (#T8070) | Beijing, CN |
| TriQuick Reagent | Thermo Scientific (#R1100) | Massachusetts, US |
| RevertAid First Strand cDNA Synthesis Kit | Thermo Scientific (#K1622) | Massachusetts, US |
| BCA Protein Assay Kit | Solarbio (#PC0020) | Beijing, CN |
| 10×Tris-Buffered Saline with Tween®-20 | Solarbio (#T1081) | Beijing, CN |
| Clarity™ Western ECL Substrate | Bio-rad (#1705060) | California, Us |
| Bovine Serum Albumin | Beyotime（#ST023） | Shanghai, CN |
| SYTOX™ Green | Thermo Scientific (#S7020) | Massachusetts, US |
| Membrane Regeneration Buffer | Solarbio (#SW3021) | Beijing, CN |
| Reactive Oxygen Species Assay Kit with CM-H2DCFDA | Beyotime (#S0035S) | Shanghai, CN |
| **Antibodies** | **Company name (catalog number)** | **City/State, Country** |
| Anti-CiTH3 | Abcam (#ab281584) | Cambridge, UK |
| Anti-NE | Abcam (#ab254178) | Cambridge, UK |
| Anti-MPO | Proteintech (#22225-1-AP) | Wuhan, CN |
| Anti-Phospho-PKC (pan) (βII Ser660) | Cell Signaling Technology (#9371T) | Massachusetts, US |
| Anti-pan-PKC | Proteintech (#12919-1-AP) | Wuhan, CN |
| Anti-phosphor-PI3K | Thermofisher (#PA5-104853) | Massachusetts, US |
| Anti-PI3K | Proteintech (#60225-1-Ig) | Wuhan, CN |
| Anti-phosphor-Raf | Hua bio (#ET1612-87) | Hangzhou, CN |
| Anti-Raf | Hua bio (#ET1710-21) | Hangzhou, CN |
| Anti-phosphor-AKT | Cell Signaling Technology (#8200S) | Massachusetts, US |
| Anti-AKT | Cell Signaling Technology (#9272S) | Massachusetts, US |
| Anti- phosphor-MEK | Yeasen (#YP-Ab-14836) | Guangzhou, CN |
| Anti-MEK | Yeasen (#YP-Ab-14836) | Guangzhou, CN |
| Anti- phosphor-ERK | Cell Signaling Technology (#4370T) | Massachusetts, US |
| Anti-ERK | Cell Signaling Technology (#4695T) | Massachusetts, US |
| Anti-PAD4 | Proteintech (#17373-1-AP) | Wuhan, CN |
| Anti-HK2 | Proteintech (#22029-1-AP) | Wuhan, CN |
| Anti-HK3 | Proteintech (#66974-1-Ig) | Wuhan, CN |
| Anti-LDHA | Proteintech (#19987-1-AP) | Wuhan, CN |
| Anti-PKM2 | Proteintech (#15822-1-AP) | Wuhan, CN |
| Anti-PDK3 | Proteintech (#12215-1-AP) | Wuhan, CN |
| Anti-GAPDH | Proteintech (#60004-1-1g) | Wuhan, CN |
| APC-Cy7-conjugated anti-CD45 | Thermo Scientific (#MA5-38731) | Massachusetts, US |
| PE-conjugated anti-CD11b | Thermo Scientific (#12-0118-42) | Massachusetts, US |
| FITC-conjugated anti-CD15 | Thermo Scientific (#11-0159-41) | Massachusetts, US |
| Goat anti-Mouse Secondary Antibody, Alexa Fluor^TM^ 647 | Invitrogen (#A-21235) | California, Us |
| Goat anti-Rabbit Secondary Antibody, Alexa Fluor^TM^ 488 | Invitrogen (#A-11008) | California, Us |
| Donkey anti-Goat Secondary Antibody, Alexa Fluor^TM^ 555 | Invitrogen (#A-21432) | California, Us |
| Goat anti-rabbit secondary antibody | Proteintech (#SA00001-2) | Wuhan, CN |
| Goat anti-mouse secondary antibody | Proteintech (#SA00001-1) | Wuhan, CN |

**Table S2** Primer sequences for qPCR

| **Gene** | **Forward prime (5′-3′)** | **Forward prime (5′-3′)** |
| --- | --- | --- |
| β-actin (human) | TGCGTGACATTAAGGAGAAGC | GGAAGGAAGGCTGGAAGAGT |
| TNF-α (human) | GCCTCGCCCTTTGCTTTACT | CTGTGGGTCTCAGGGAGATCA |
| IL-1β (human) | GACAGGATATGGAGCAACAAGT | TCAACACGCAGGACAGGTA |
| IL-6 (human) | TTCTTGGGACTGATGCTGGTGAC | CTGTTGGGAGTGGTATCCTCTGTG |
| CCL2 (human) | CTCGCGAGCTATAGAAGAATCAC | CATGGAATCCTGAACCCACTT |
| CXCL10 (human) | CCCAAGTGCTGCCGTCATTTTC | TAGGCTCGCAGGGATGATTTCAAG |
| IL-10 (human) | GACTTTAAGGGTTACCTGGGTTG | TCACATGCGCCTTGATGTCTG |

**Supporting figures**


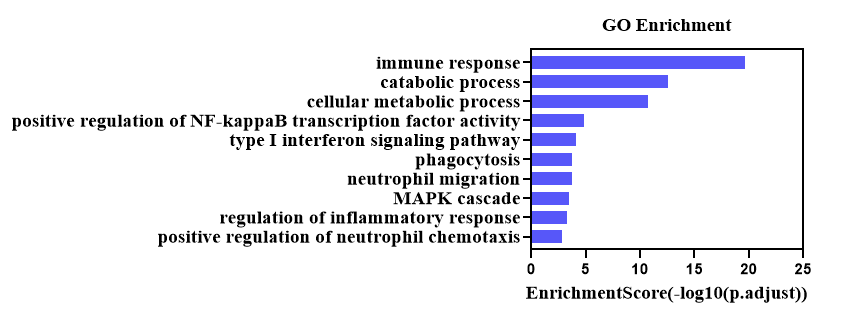


**Figure S1** The GO pathways enriched with differentially expressed pathways between control and IFNα2 groups.


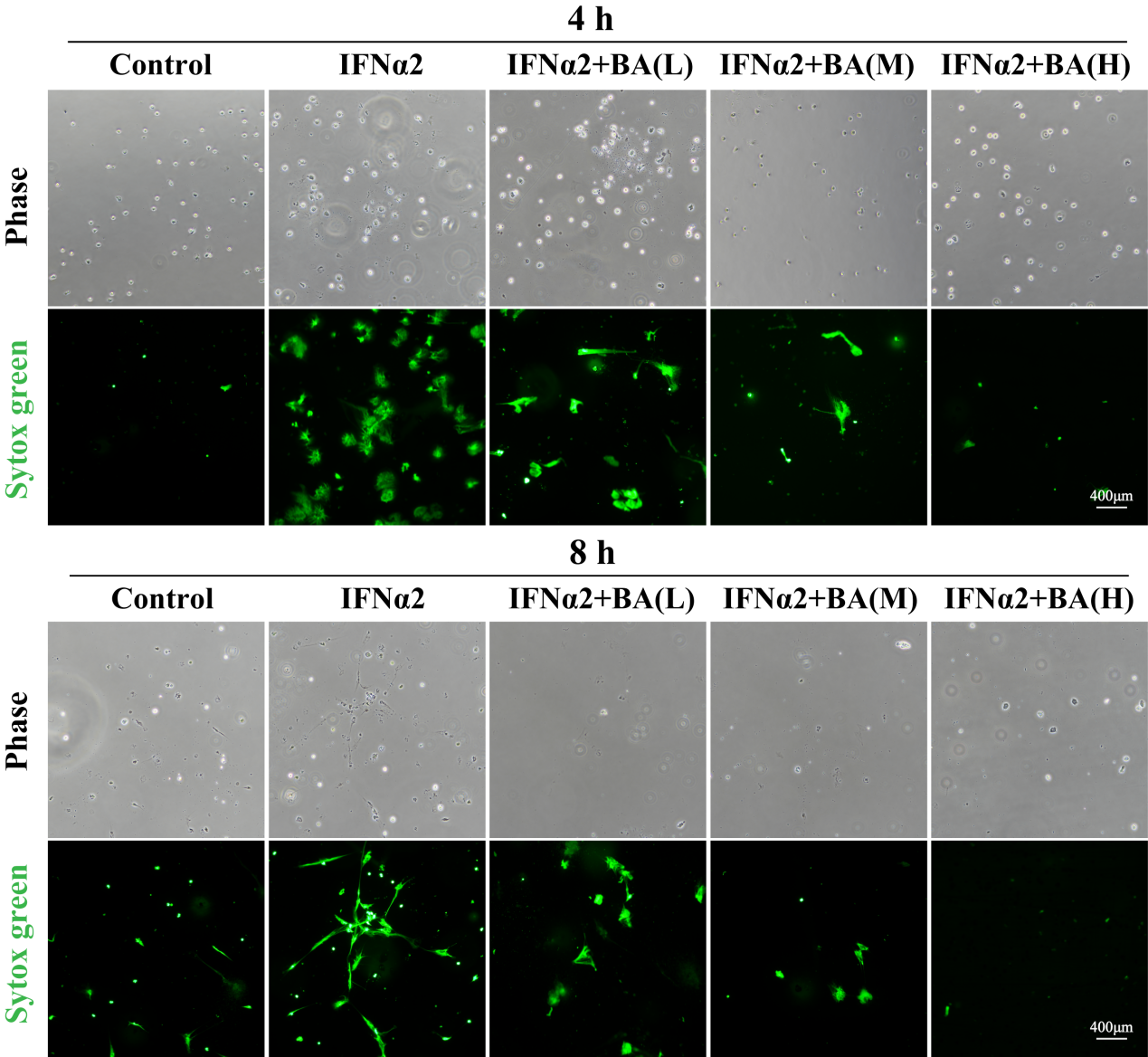


**Figure S2** Fluorescence images of NETs were quantified using the cell-impermeable nucleic acid stain Sytox Green. Scale bars, 400 μm.


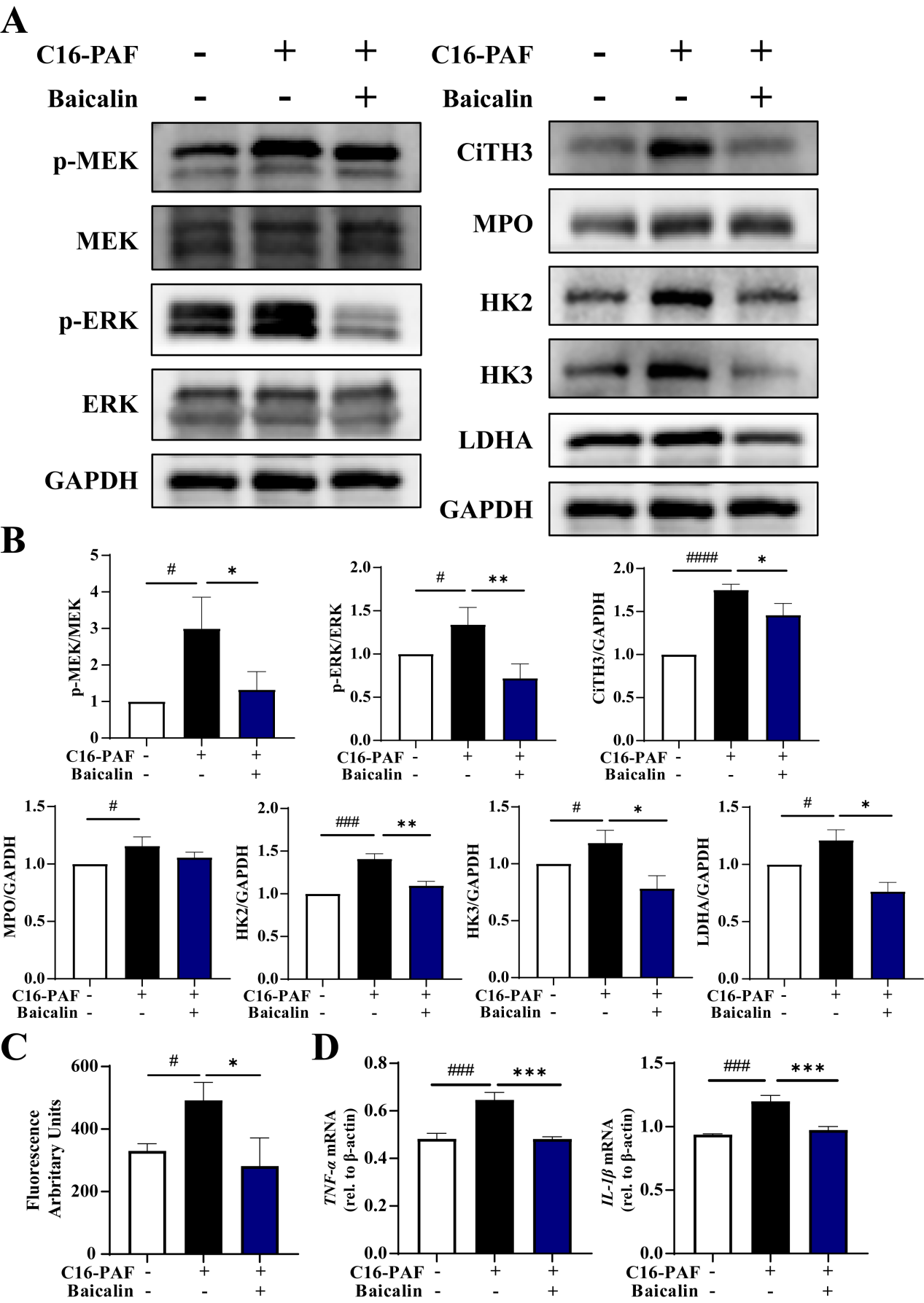


**Figure S3** Baicalin exhibited inhibitory effects on neutrophil NETs and glycolysis levels induced by C16-PAF. The neutrophils were treated with the ERK agonist C16-PAF for 1 h before Baicalin treatment. (A-B) The proteins expression levels of p-MEK, MEK, p-ERK, ERK, Cith3, MPO, HK2, HK3, and LDHA were detected using Western blotting (A) and relative quantitative analysis (B). (C) The fluorescence of NETs stained with cell-impermeable Sytox Green was measured using an excitation wavelength of 504 nm and an emission wavelength of 523 nm. (D) The mRNA levels of inflammatory cytokines *TNF-α* and *IL-1β* were detected by qRT-PCR. Data are shown as mean ± SD, #*P* < 0.05, ###*P* < 0.001, ####*P* < 0.0001 vs. Control group; **P* < 0.05, ***P* < 0.01, ****P* < 0.001 vs. IFNα2 group.


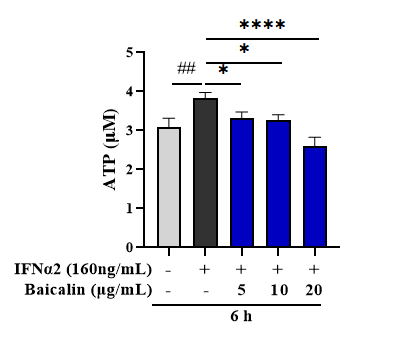

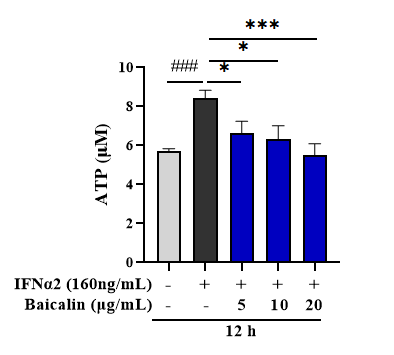


**Figure S4** The neutrophils were stimulated by IFNα2, with or without treatment of Baicalin (5, 10, 20 μg/mL), for 6 h and 12 h respectively. Subsequently, the levels of ATP in neutrophils were measured using ATP assay kit respectively.


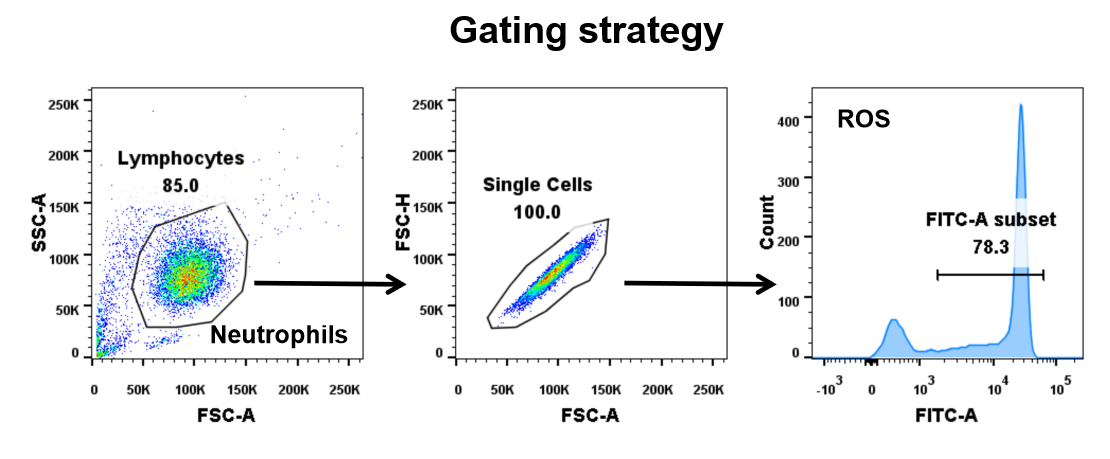

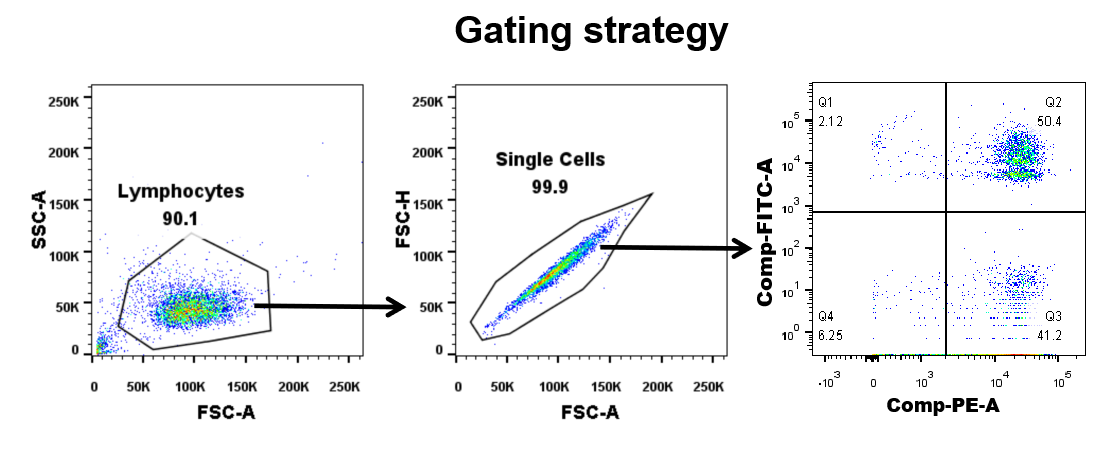


**A**

**B**

**Figure S5** (A-B) Gating strategies in flow cytometry for neutrophil ROS and phagocytosis function analysis.
